# Endometrial Receptivity Analysis (ERA) test: an unproven technology

**DOI:** 10.1093/hropen/hoab010

**Published:** 2021-04-14

**Authors:** Zion Ben Rafael

**Affiliations:** Adelson School of Medicine, Ariel, University, Israel

**Keywords:** ERA test, add-ons, ART, IVF, implantation failure, RIF

## Abstract

This article addresses the limitations of the endometrial receptivity array (ERA) methodology to increase implantation. Such limitations vary from the assumed inconsistency of the endometrial biopsy, the variable number of genes found to be dysregulated in endometrium samples without the embryonal-induced effect, the failure to account for the simultaneous serum progesterone level, and the expected low percentage of patients who may need this add-on procedure, to the difficulties in synchronising the endometrium with hormone replacements in successive cycles and the inherent perinatal risks associated with routine cryopreservation of embryos. Without a gold standard to compare, the claim that the window of implantation (WOI) might be off by ±12 h only requires a good argument for the advantage it provides to human procreation, knowing that embryos can linger for days before actual embedding starts and that the window is actually a few days. The intra-patient variations in the test need to be addressed. In summary, like all other add-ons, it is doubtful whether the ERA test use can significantly enhance implantation success rates.

## Introduction

The endometrial receptivity array (ERA) test is a new player amongst the plethora of add-ons ‘solutions’ for repeated implantation failure (RIF), but it seems to be following the same path as other add-ons which were deemed unproven due to failure to demonstrate an increase in success rate, mainly the live birth rate (LBR). Using an invasive biopsy of the endometrium (a sort of scratching with the help of Pipelle) at the time of the presumed window of implantation (WOI), the ERA test attempts to evaluate the gene expression profile, with the hope of identifying the specific transcriptomic signature, and offering a more precise timing for personalised embryo transfer (pET).

The ERA test divides the results of the endometrial biopsy into receptive and non-receptive, and further to pre-receptive or post-receptive, by using very complex statistics (Ruiz-Alonso, 2013, 2014; Garcia-Velasco, 2015*;*Mahajan, 2015) with the intention of freezing all embryos for future transfer while enhancing or delaying the WOI artificially, according to the results.

Offering a diagnostic test to better synchronise the embryo and the uterus is compelling, however, there are many questions that need to be answered before considering the clinical results of the first RCT that was recently published (Simón *et al.*, 2020). The ERA test was offered commercially as a successful tool to increase precision of the WOI in presumed RIF, beyond the ineffective histological dating (Díaz-Gimeno *et al.*, 2013), when it was only in the early stages of exploration and before real proofs were presented to indicate an increase in IVF success. In addition, in the original case-study, only 17 patients were included, of whom 10 had one to two failures, 4 had three failures and the remaining 3 had more than three failures (Ruiz-Alonso *et al.*, 2013). This was hardly a population of RIF by any definition (Ben Rafael, 2020a). It is noteworthy that we have recently condemned RIF as an invalid diagnosis to denote ‘failure to implant’, since no two failures are similar, and failure might be due to completely different reasons each time. The idea that several failures can be allocated under one name (RIF) and receive special attention and treatment, when not is really an iatrogenic entity (Somigliana *et al.*, 2017; Ben Rafael, 2020a), was also denounced in an editorial in Human Reproduction (Evers, 2016).

The ERA test raises multiple questions which were not answered by the RCT. First, the array methodology has now largely been replaced by next generation sequencing (NGS). Second, how many genes should be included in the test? Many groups using microarray have found a different number of genes that were up or down regulated during the WOI. This, by itself, hints at the volatility and dependency of the results on the methods used to test the genes, the complexity of the mathematical model, the methods and timing of biopsy, corrective methods to the biopsy material, relationships to preovulatory progesterone levels (which are overlooked in most studies) and the validity of displacing the WOI in successive cycles by only 12–24 h. In addition, the implantation process includes crosstalk between the endometrium and the embryo before and during invasion (Diedrich *et al.*, 2007). This includes many distinct embryonal stages like apposition, adhesion and invasion which are regulated by many genes over a restricted period of days. The biopsies for gene expression evaluation are done on an endometrium that has not been affected by the embryo-endometrial crosstalk, which represents an obvious limitation of the whole concept. Furthermore, any hormonal manipulation of the WOI in subsequent cycles can affect gene expression differently, which may result in high rates of intra-patient variability in repeated testing on the same patients.

## Number of genes tested

A partial list of gene numbers that were found to be dysregulated at the WOI varies from 63 genes (Tapia *et al.*, 2008), 238 genes (in commercial ERA test, Ruiz-Alonso *et al.*, 2013, recently dropped to 236 genes), 303 genes (Macklon, 2017) and 616 genes that were upregulated (Huang *et al.*, 2017); to 313 genes of which 92% were down and 8% up-regulated (Koler *et al.*, 2009). Others have found 91 genes significantly increased more than 2-fold in their expression, and 115 were decreased more than 2-fold in endometriosis patients (Kao *et al.*, 2003). Furthermore, earlier studies by the same group behind the commercial test were also different. They found that 147 genes were significantly dysregulated in the refractory endometrium (Horcajadas *et al.*, 2006) or 218 and 133 genes that changed on day hCG + 7 versus LH + 7 accordingly (Horcajadas *et al.*, 2008), compared to the current 238 genes in the ERA test. The last number already hints that gene profile at LH + 7 is not comparable to hCG + 7 as maintained by the ERA test.

The different number of genes here and in other publications is not easy to reconcile, and therefore requires a sophisticated statistics analysis to form a clinical conclusion (Garcia-Velasco *et al.*, 2015). They are not self-explanatory for the clinician’s (or client’s) judgment, bearing witness to the randomity of the search for a credible signature of the receptive, pre-receptive or post-receptive endometrium. This issue cannot be separated from the question of consistency of the methods used and the repeatability of the test in the same patients. Cho *et al.* (2018) have shown a large intra-patient variability in the results of gene profiles which might be due to the methods used or the hormonal status.

The same group (Garcia-Velasco *et al.*, 2015) had their own doubts on the issue. In a study of endometrium in endometriosis patients, it was claimed that ‘endometrial receptivity’ is a multifactorial process of which the studied gene expression is but one factor, as ‘other genes that may not have been studied, epigenetic aberrations or even pathologic proteomic profiles might provide further insight’. Unexpectedly, women with endometriosis who were postulated to have an endometrial receptivity defect and progesterone resistance (Fox *et al.*, 2016), did not differ in their ERA test results (Miravet-Valenciano *et al.*, 2017).

To add to the confusion, it is maintained that it is unlikely that a single endometrial cause underlies RIF (Macklon, 2017) since most failures are due to the embryo quality (Diedrich *et al.*, 2007), and studies show that after 3–4 IVF cycles, only a few women remain not pregnant. This notion supports the idea that a faulty endometrium is infrequently the cause of RIF (Ben-Rafael, 2020a). Also, it has been shown that RIF is not associated per se with abnormal endometrial integrin expression, a marker of implantation, or the expression of endometrial integrins a1, a4 and aVb3, which appear to have no prognostic value in subsequent IVF treatments (Coughlan *et al.*, 2013).

Given that various pathologies have been found in endometrial biopsies (Crum *et al.*, 2003), and that ‘histological dating alone lacks the sensitivity to identify a definable defect in endometrial development and, therefore, in the implantation process’, Kliman and Frankfurter (2019) claimed that dating is not the reason for implantation failure.

## Array versus sequencing

Technology is evolving fast. It was recently claimed that all the past and current studies using array technology might not be accepted as accurate anymore. ‘It is now well accepted that sequencing technique NGS is more comprehensive in coverage and precise in quantification of global gene expression profiles’ (McGettigan *et al.*, 2017; Huang *et al.*, 2017). Hence, all the studies on the ERA, including the recent RCT (Simón *et al.*, 2020), which have used array test become difficult to interpret, and should be re-evaluated based on NGS.

## Method and timing of biopsy and endometrial correction

Can a ‘blind’ manual procedure of endometrial cells collection be standardised to derive each time consistent and representative cell populations? Pipelle, depending on the operator, may result in variable amounts and depth of cell collection, hence it is not surprising that the group that developed the test has recently recognised this issue and provided a correction that identifies the relative contribution of the epithelial and stromal cells in the biopsy to the gene expression profile. They have offered a partial solution by using ‘computational deconvolution’ which is a statistical, mathematical correction method, to evaluate the relative contribution of the major cell types to the transcriptome, but not of the less frequent cells (Suhorutshenko *et al.*, 2018) which create another limitation of the newly offered corrective method. It should be noted that ‘computational deconvolution’ was not used in the RCT which started before its introduction.

The timing of biopsy in relationship to the WOI should also be questioned. It was proven that fertile and infertile women had similar out of phase biopsies (Coutifaris *et al.*, 2004), hence without a gold standard for the exact implantation window, the timing of the biopsy (i.e. the WOI) remains controversial. If we cannot consistently identify the ovulation (Park *et al.*, 2007; O’Connor *et al.*, 2006), then the timing of biopsy and gene profile might be skewed accordingly. For example, while recent reviews have found that the optimal day of embryo transfer was LH + 6 or hCG + 7 (Mackens *et al.*, 2017), the ERA test considers LH + 7 and hCG +7 (Ruiz-Alonso *et al.*, 2013) or hCG +7 (Horcajadas *et al.*, 2006) as equal and optimal. Others also rejected the idea that LH+ 7 is equal to P + 5 and claimed that defining the date of biopsy is not simple and that it is inconsistent in natural and medicated cycles (Kliman and Frankfurter, 2019). Furthermore, it should be noted that any endometrial hormonal manipulation before embryo transfer (ET) or any ovarian stimulation or triggering of ovulation may affect the gene profile differently (Horcajadas *et al.*, 2008; Humaidan *et al.*, 2012; Mahajan, 2015).

## Progesterone effect

Progesterone is the undeclared elephant in the room. Progesterone is the main driver of the secretory changes that determine the WOI and there are complex correlations between progesterone, implantation and the success rate, and yet the ERA test was presented independently of progesterone levels. Progesterone starts to rise slightly before ovulation and a day before oocyte retrieval because of exogenous hCG. This can vary with the number of follicles. Progesterone alters gene expression (Fatemi and Van Vaerenbergh, 2015). A preovulatory rise in progesterone above 1.5 ng/ml has been associated with lower pregnancy rates (Bosch *et al.*, 2010) and dysregulation of over 140 endometrial genes that are required for normal endometrial function (64 up- and 76 down-regulated) while 13 marker genes of receptivity were over regulated (Labarta *et al.*, 2011). This dictates that any new procedure that is based on gene expression, should consider the concomitant progesterone level.

In yet another study, it was proposed that progesterone serum levels of less than 10 ng/ml on the day of embryo transfer are associated with a lower success rate (Labarta *et al.*, 2017) that is fully correctable by the addition of progesterone in the same cycle (Labarta; presented during COGI Paris 2019; ww.congresmed.com/COGI). Additionally, others have suggested that ‘P levels of >5 ng/ml that act on an adequately primed endometrium result in endometrial luteinisation and receptivity, which does not differ from that achieved by much higher levels’ (Usadi *et al.*, 2008; De Ziegler *et al.*, 2013). However, progesterone levels were not reported in conjunction with gene profile studies, but rightfully in the RCT, patients with a preovulatory progesterone rise were excluded.

When planning to correct a pre-receptive or post-receptive endometrium, we need to freeze all embryos and rely on hormone replacement cycles, but neither the serum progesterone levels that are required to optimise cycle outcome, nor the optimal length of exposure to progesterone before frozen embryo transfer, have been firmly established (Van de Vijver *et al.*, 2016). In the ERA test, 5 days of progesterone administration is considered optimal for implantation of Day 5 embryos, and progesterone exposure is assumed to be able to enhance or delay the endometrial maturity by precisely ±12 h intervals. This idea is not supported by studies which have shown that plus or minus one day in progesterone exposure does not affect implantation. Van de Vijver *et al.* (2016) randomised two groups who received a Day 4 embryo transfer after 3 or 5 days of progesterone exposure. The pregnancy rate was similar in both groups, although a shorter progesterone exposure was associated with a higher miscarriage rate. So ± 12–24 h was not shown to be a problem regarding implantation, which raises a disagreement over the question of ‘what difference does one day make?’ (Ruiz-Alonso *et al.*, 2014). Furthermore, the feasibility of manipulating the endometrium with such precision has not been proven, suffice to say the variability in the compliance of the ‘progesterone start’ between patients might span more than 12 h. Kliman and Frankfurter (2019) rejected the idea that LH + 7 is equal to P + 5 and questioned why such precision in embryo transfer is critical given that the implantation window is at least 3 days in duration.

As for luteal support, serum P levels vary widely even when the same preparations are given, since the uptake, absorption and metabolism of each hormone varies amongst women (Yovich *et al.*, 2015). Also, intravaginal progesterone is thought to have a first pass and effect on the endometrium that is beyond the serum levels (Cicinelli *et al.*, 2001), but neither serum progesterone levels nor endometrial tissue concentrations seem dose proportional (Paulson *et al.*, 2014). Taken together these data indicate that it is not so feasible to manipulate (enhance or delay) the endometrium in the coming cycle by relying only on the number of hours or days of progesterone administration to displace the WOI by ±12–24 h.

## What percentage of women might need the ERA test?

The repeated claim that about 25% of failed IVF cycles are due to endometrial problems is not supported by their own study. According to Mahajan (2015), the ERA test is probably applicable to a marginal number of patients. They have used the test in a group 186 patients, dividing them into two groups: Group 1 who failed only one IVF cycle and were found to have 15.1% nonreceptive endometrium, and Group 2 who failed three IVF cycles and were found to have 27.5% non-receptive endometrium. The non-receptive cases underwent a 12 h (only) modification/correction of the WOI in the subsequent thawed cycle. The only pregnancy registered after one previous failure and correction of the non-receptive endometrium miscarried (1/7 i.e. 14.3%). However, after three failures, the correction of the non-receptive endometrium resulted in a 44.5% (8/18) pregnancy rate of which one was aborted, an apparent good result. Calculated differently, the overall pregnancy rate in the first cycle was 61%, and in the third cycle before any corrections were made, it was 42%. Assuming that in the second cycle (which was not reported) the pregnancy rate was also about 40%, it leaves us with only about 6.5% women not pregnant after three failed cycles, of whom 27.5% were non-receptive (a similar percentage of non-receptive endometrium was suggested after three failures also by others; Hashimoto *et al.*, 2017). which is about 1.8% of all patients treated who can potentially utilise the test following three implantation failures. Even with a lower pregnancy rate of 30–35% in each cycle for three cycles, the test is applicable to only 5–10% of all patients starting, hardly a breakthrough considering that even after three failures the chances of conceiving are still very good. A confirmation that RIF (three IVF failures) is rare, as is the need for changes including the ERA test, was provided in a recent study (Pirtea *et al.*, 2020) on 4428 patients who received a thawed euploid single embryo transfer (SET). The cumulative pregnancy rate after three cycles was 95.2% and the LBR was 92.6%, which supports our above calculation (Mahajan, 2015) that the ERA test, if done after three failures, will apply only to a minority of IVF failures.

## A recent multicentric RCT

RCTs, and even meta-analysis on the different add-ons, have initially shown positive or at least encouraging results, and only when they ae used on a larger scale or with the correct design, questions and planning, have their true value emerged (Ben Rafael, 2020a).

Unlike the seminal non-controlled studies on the ERA test, the recent multicentric open label RCT (Simón *et al.*, 2020) did not deal with RIF. They aimed to compare pET to fresh or frozen ET in younger age women, under 37, in their first IVF cycle (about 70%) or after a few failures (30%); these are clearly not cases that needed experimental add-ons procedures. For a multicentric study, many issues in the protocol, such as stimulation protocol, vitrification method and progesterone dose in the transfer cycle were not fully controlled and left to the decision of the participating centre, a fact that must have created an heterogeneity in the study group. Also, with the current trend to substitute array test with more precise NGS technology, all previous studies including the RCT, with the old version of array gene expression should be re-confirmed by NGS (McGettigan *et al.*, 2017; Huang *et al.*, 2017).

It is beyond the scope of this paper to provide a critical review of the study design, exclusion and inclusion criteria, before and after oocyte collection, but it is important to indicate that by running the ERA test only on the pET group and not on the control groups, they missed the chance to comment on the most important issue, i.e. what can be expected when women with a non-receptive endometrium or ‘positive ERA test’ keep trying in the successive cycles without any ‘correction’?

Nevertheless, the results of the primary outcome measured in this RCT showed no differences in pregnancy rate or LBR by intention-to-treat analysis (an unbiased estimate of the efficacy of the intervention) or by first embryo transfer. Only the cumulative LBR after 12 months was higher.

A point of caution stems from the fact that despite the exclusion of all bad-risk patients and patients with non-satisfactory stimulation and high preovulatory progesterone, the percentage of women with displaced WOI (before any failure) was 37.5%, which is higher than the previously reported 27.5% after three implantation failures or 15.5% after one failure (Mahajan, 2015; Hashimoto *et al.*, 2017).

## Ill-effect of embryo cryopreservation

Finally, the freezing embryos for later transfer without a good indication, may be wrong (Ben-Rafael, 2020b). The significantly higher rate of preeclampsia (7.5% vs. 4.9%) and eclampsia (4% vs. 2.5%), including chronic hypertension after frozen-thawed embryo transfers and the rates of premature labour after the use of frozen oocytes compared to fresh, are arguments accumulating against freezing all embryo (Sites *et al.*, 2017). A 3-fold increase in hypertensive disorders (4.4% vs. 1.4%; *P* < 0.009) after freezing was also confirmed in PCOS patients (Chen *et al.*, 2016). Both hypertensive disorders and large for gestational age were confirmed in a meta-analysis (Maheshwari *et al.*, 2018).

## Summary and discussion

A complex crosstalk between the endometrium and the embryo during implantation includes genes expression and anatomical, physiological and metabolic changes, all of which can vary with the medical treatment administered. We are far from being able to test for or having a full understanding of these processes, hence, every measurement of one such effect might represent a tubular rather than perspective outlook. Since most IVF failures are due to the quality of the embryos, focusing on the endometrium as a reason for IVF failure is probably applicable in only a minority of cases.

The hurdle of the ERA test, to prove the existence of a non-receptive endometrium, and pinpoint the displacement within a few hours frame, assumes that progesterone manipulation can bridge a ± 12–24 h gap, and correct the endometrium, without having a gold standard to compare to any of these effects, all with a single tool namely gene profiling, seems insurmountable. For example, it has long been maintained that ovarian stimulation cycles result in an asynchronous endometrium, which obviously calls for different measures including ‘freeze all’ to circumvent the ‘problem’, but the problem of asynchronous endometrium has never been shown to exist and it is doubtful whether it needs any correction, except in special cases (Ben Rafael, 2020b).

It is not surprising that the only RCT that was published so far (Simón *et al.*, 2020) could not show, based on intention to treat, any improvement. Furthermore, another recent retrospective study by the same group (Cozzolino *et al.*, 2020) has concluded that the use of the ERA test in 488 patients who underwent preimplantation genetic testing for aneuploidy (PGT-A), ERA or both, could not show any advantage to the use of ERA test. In short, ERA did not appear to improve outcomes in a group of patients who failed three or more ETs with a total of three or five embryos, and strangely PGT-A per se was effective in those failing three ETs, but was ineffective after failures of transfer of five embryos (Cozzolino *et al.*, 2020). Similarly, others (Neves *et al.*, 2019) in a multivariate analysis have confirmed that performing an ERA test did not influence the pregnancy rate, in the tested euploid ET arm and was even associated with a diminished pregnancy rate in the donor ET arm. Taken together, these observations coupled with the lack of intra-patient consistency, bare witness to the randomity of the current state of research in the field.

Different studies have documented a 10-fold spread (from 63 to 616) in the number of genes that are dysregulated at the WOI, which casts doubt on the meaning of any number of genes in indicating a correlation with implantation. This might be partly explained by the array method, which is no longer accepted as accurate and should be replaced by NGS, and the blind method of biopsy which might be hard to standardise to provide similar cell populations every time. These issues need more research and awareness. Furthermore, without bringing into the equation the different factors that can potentially affect implantation, such as oestrogen priming, progesterone type and route of administration including local and peripheral levels, and the changes they induce on the endometrium, the gene expression signature by itself, even with NGS, cannot reliably reflect the full complexity of the implantation process.

The idea of improving implantation through high technology is most welcome, but like any new procedure, it must be reconciled with several known facts and be proven beyond any doubt before it is widely offered commercially. The suggestion that the endometrium may be delayed or enhanced at the WOI, by as little as ±12 or 24 h (Mahajan, 2015; Valdes *et al.*, 2017; Simón *et al.*, 2020) almost implies a ‘point of implantation’ rather than a ‘window of implantation’ and requires a good argument for the advantage it provides to human procreation, rectified with the known facts that embryos can be transferred once or twice (double ET) in any of the first 6 days and can linger for days before the actual embedding starts, thus showing a high degree of tolerance to a non-receptive endometrium before it turns to be receptive. We also know that two embryos can implant in the same uterus days, or even weeks, apart (superfetation or superfecundation).

Clinicians tend to grasp any new idea that can potentially improve results (Ben Rafael, 2020a), also to demonstrate that they are competitive and updated, but unfortunately even after many years of practice, most add-ons have been deemed not proven (Macklon *et al.*, 2019). New tests, like the ERA, until shaped and proven, should be offered only under research protocols that separate compounding factors, considering all the above reservations, and keeping in mind that freezing embryos is not risk free. As we have witnessed time and again, a single RCT or even a meta-analysis should not be accepted as a final proof towards its overall utility as a new solution or its wide-spread use (Ben Rafael, 2020a). It is the duty of the clinical societies and peer review journals to follow up on the evidence, and filter new ideas and technological procedures, to avert physicians from repeating false directions or mistakes for longer than necessary.

As physicians, it is our continuous duty to appraise any new treatment and diagnostic tool and offer an add-on procedure only by indication and if it is proven to provide a better LBR under research protocols. The message to the patients should be that failures occur more often than not, and if no special obstacle to pregnancy exists, when stimulation, embryo culture and endometrial width appear to be normal, there is no need to resort to unproven costly add-ons, and, if patients agree, they need to persevere with their similar trials for five or more cycles, which will leave only a small fraction of patients in need other, albeit unproven, solutions.

## Data availability

No new data were generated or analysed in support of this research.

## Funding

The author declares no funding was given to this work.

## Conflict of interest

The author declares no conflict of interest. Author has written and reviewed the manuscript.
